# Ludwig's Angina—A Controversial Surgical Emergency: How We Do It

**DOI:** 10.1155/2011/231816

**Published:** 2011-07-06

**Authors:** Wael Hasan, David Leonard, John Russell

**Affiliations:** ^1^Otolaryngology/Head & Neck Surgery Department, University College Dublin, Dublin 4, Ireland; ^2^Otolaryngology/Head & Neck Surgery Department, St. Vincent's University Hospital, Dublin 4, Ireland

## Abstract

*Objectives*. To review the current protocols used for management of Ludwig's angina and to assess the efficacy of conservative measures in these cases. *Methods*. A retrospective review of patients who were admitted to our institution for management of Ludwig's angina between 2003 and 2010. *Results*. Two patients were identified. Both were managed successfully with conservative measures and close airway observation. None needed an emergency intubation or surgical tracheostomy. There were no mortalities, and both had a short hospital stay. *Conclusion*. Recently, management of Ludwig's angina has evolved from aggressive airway management into a more conservative one. This is based on close airway observation on a specialised airway unit and a serial clinical airway assessment. Improved imaging modalities, antibiotic therapy, surgical skills, and clinical experience are the key factors behind this change in practice.

## 1. Introduction

Ludwig's angina is a known, yet a rare surgical emergency that is potentially life threatening unless early recognised and aggressively treated. Airway management is the main foundation in these cases. Despite that, no specific guidelines exist and management is largely dependent on clinical judgment and experience. Many controversies regarding different conservative and surgical management options exist. In this paper we highlight these controversies with a brief review of the literature and a retrospective review of recent experiences with Ludwig's angina.

## 2. Case  1

A 56-year-old male presented with a 72-hour history of a worsening dysphagia and submandibular swelling. The onset was following a visit to the dentist where a dental abscess was drained. 24 hours later he revisited his dentist complaining of a submandibular swelling, and was commenced on oral Co-amoxiclav. Despite that, his symptoms progressed and he made his way to our facility 72 hours later. Based on his history and symptoms the diagnosis of Ludwig's angina was suspected. He was febrile of 38.1°C and drooling. He was not in respiratory distress; his respiratory rate was 23, and O_2_ saturation was 96% on room air. Oral cavity exam showed mild trismus and inflamed floor of mouth. Fiberoptic laryngoscopy was performed and showed a patent laryngeal airway and a normal epiglottis. Based on this initial assessment, the decision was to manage him conservatively with close airway observation. He was commenced on intravenous Co-amoxiclav and Clindamycin, and a contrast-enhanced CT scan of his neck and upper thorax was performed to rule out the possibility of a deep neck abscess. This showed an extensive submandibular soft tissue swelling and inflammation with moderate tongue elevation and posterior displacement causing a degree of oropharyngeal airway compromise. No abscess cavity or laryngeal airway compromise was evident. He was admitted to the ear, nose and throat (ENT) ward for hourly airway observation and intravenous antibiotic treatment. His symptoms improved on a daily basis maintaining a normal breathing rate and pattern with no O_2_ desaturations below 97% on room air on pulse oximetry. By the 5th day following his admission, his symptoms have fully resolved, and he was discharged from hospital on oral Co-amoxiclav. Two weeks and 3 months out-patient department followups showed no recurrent soft tissue swelling or oedema, and he was discharged from our service.

## 3. Case  2

**Figure 1:**
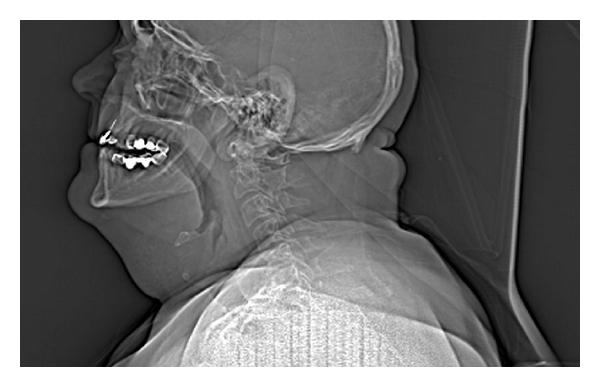
A scout CT view of the patient in case 2. This view shows a patent oropharyngeal airway despite superior and posterior displacement of the tongue secondary to a significant submandibular inflammation and swelling.

**Figure 2:**
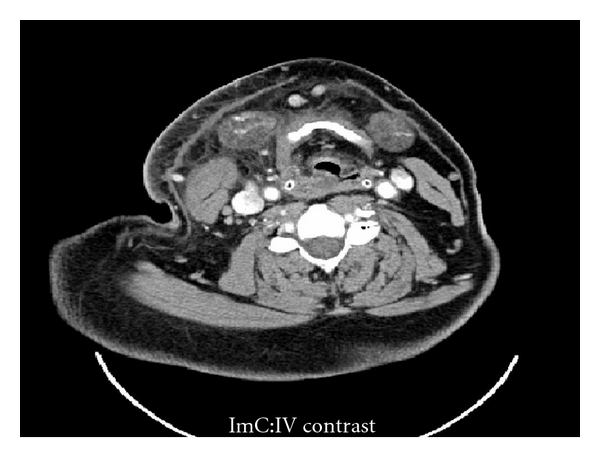
Axial contrast-enhanced CT scan of the patient in Figure 1 showing diffuse swelling of the right submandibular region, stranding of the cervical fat, thickening of the right sternocleidomastoid muscle and the right pyriform fossa. The laryngeal airway is mildly effaced and displaced superiorly.

A 59-year-old male with a background history of noninsulin-dependant diabetes mellitus and sebopsoriasis presented with 24-hour history of mild right-sided facial swelling, a worsening submandibular swelling, and mild dysphagia. He had low-grade pyrexia of 37.8°C, his respiratory rate was within normal limits, and his O_2_ saturation was 98% on room air. On physical examination he had a mild right-sided facial swelling, a significant submandibular swelling, and skin erythema extending inferiorly to the level of the upper border of the sternum. A fiberoptic laryngoscopy showed a mild oropharyngeal narrowing and a patent laryngeal airway. He was commenced on Intravenous Clindamycin, Ciprofloxacin, and Benzylpenicillin, and a contrast-enhanced CT scan of his neck and upper thorax (Figures 1 and 2) was performed to role out a deep neck abscess or a mediastinum extension. This showed a significant submandibular soft tissue inflammation and oedema with a degree of oropharyngeal compromise. His laryngeal airway was normal, and no deep neck abscess or fascial extension was evident. He was admitted to the ENT ward for hourly airway observation and intravenous antibiotic treatment.

On the third day after admission, a mild improvement of his submandibular swelling was evident. On the other hand, a worrying inferiorly spreading skin erythema to the level of the 5th costal cartilage was noted. Despite that, his respiratory rate and O_2_ saturation remained unchanged, and he had no cardiothoracic complaints to suggest a danger space extension or a necrotising fasciitis. A clinical assessment including a chest plain film and a neck ultrasound was performed. No evidence of a deep neck abscess or a necrotising fasciitis was found. His intravenous antibiotic combination was changed to Clindamycin, Ciprofloxacin, and Teicoplanin. The dermatology service was consulted, and a secondary psoriatic flare up was suspected. A topical Daktacort–Hydrocortisone cream (Miconazole and Hydrocortisone) was added. A gradual improvement of his symptoms occurred on daily basis. By the sixth day following his admission his symptoms have fully resolved, and he was discharged from hospital. Two weeks and 3 months out-patient department followups showed no recurrent laryngeal oedema or skin erythema, and he was discharged from our service.

## 4. Discussion

Ludwig's angina is named after the German physician, Wilhelm Friedrich von Ludwig who first described this condition in 1836 [1]. It is a potentially life-threatening cellulitis, or connective tissue infection, of the neck and floor of the mouth which is characterised by progressive submandibular swelling with elevation and posterior displacement of the tongue [2, 3].

Odontogenic infections account for the majority of cases [4]. The most commonly cultured organisms include *Staphylococcus, Streptococcus, and Bacteroides* species [5].

Early antibiotic treatment should be broad spectrum to cover Gram-positive and Gram-negative bacteria as well as anaerobes. A Combination of penicillin, clindamycin, and metronidazole is commonly used.

The use of intravenous steroids has been proposed as a mean of reducing soft tissue swelling and oedema and minimising the likelihood for the need of a surgical airway in Ludwig's angina [1, 5, 6]. This remains controversial, as up to this date no randomised controlled trials that demonstrate the efficacy of corticosteroids in these patients exist.

Traditionally aggressive airway management by securing the airway with endotracheal intubation or surgically with a surgical tracheostomy was the norm.

Although no specific guidelines are present for managing acute Ludwig's angina, decisions regarding airway protection are largely dependant on the “Practice Guidelines for Management of the Difficult Airway” that were adopted by the American Society of Anaesthesiologists in 1992 and updated in 2003 [7]. In these guidelines, a difficult airway is defined as “the clinical situation in which a conventionally trained anaesthesiologist experiences difficulty with face mask ventilation of the upper airway, difficulty with tracheal intubation, or both.” When that is the case, patients are intubated via awake assisted fiberoptic bronchoscope. When this fails a surgical tracheostomy is performed under local anaesthesia. The guidelines specify that these recommendations may be adopted, modified, or rejected according to the clinical needs and constraints as these guidelines are not intended as standards or absolute requirements and their purpose is to assist the practitioner in decisions about health care [7].

Recent reports have encouraged conservative management of Ludwig's angina in selected patients over the conventional aggressive airway management. This includes intravenous antibiotic therapy and close airway observation. Larawin et al. retrospectively studied a total of 103 patients with deep neck space infections from 1993 to 2005. Ludwig's angina was the most commonly encountered infection seen in 38 (37%) patients of treatment. 13 (34%) patients managed successfully with medical therapy and only 4 (10%) patients required a tracheostomy tube [8]. 

Kurien et al. reported a 13-year review of patients with Ludwig's angina between 1982 and 1995. Patients were either admitted to the ENT or paediatric surgical units.

There were 41 patients, 24% being children and 76% adults. In children, 70% were controlled with conservative medical management while 81% of adults required incision and drainage. Tracheostomy was necessary in 10% of the children and in 52% of the adults. Mortality rate was 10% in both groups [9].

A 9-year review by Greenberg et al. of 29 cases of deep neck space infections reported 21 patients (72 %) treated conservatively following initial clinical assessment.

One of these patients subsequently deteriorated requiring emergency intubation. Of those treated nonconservatively at initial presentation, 7 (24%) patients were able to be intubated using fiberoptic nasoendoscopy and 1(3%) patient required tracheostomy under local anaesthesia [10].

Early detailed imaging is essential to evaluate the extension of tissue infection or necrosis and to guide decisions regarding surgical approaches when indicated. CT scan and MRI are of invaluable importance in the assessment of deep neck space infections and collections. Miller et al. [11] reported that combined clinical evaluation and CT findings lead to accuracy of 89%, sensitivity of 95%, and specificity of 80 % in identifying drainable collection. Plain chest radiographs are useful when looking for signs of mediastinum extension such as mediastinitis and pleural effusion.

Although ultrasound is not as easily interpreted by clinicians and surgeons as other imaging modalities, its availability, cost effectiveness, reduced risk of radiation, and accuracy in differentiating cellulites-related oedemas from abscess collections make it a reliable supplement modality to CT scan in resistant cases [12, 13].

However, in cases of significant airway compromise where an immediate decision regarding the need of a definitive airway is required, clinical experience and judgment are superior to imaging.

## 5. How We Do It

From our clinical experience and the literature review we conclude that conservative management of Ludwig's angina is acceptable in selective cases, provided that early antibiotic therapy is commenced and any collectable abscess is drained. We also propose an airway management protocol for these cases. In this, initial airway assessment is based on respiratory rate, oxygen saturation, and findings on fiberoptic laryngoscopy. Patients are then categorised as having either a severe airway compromise or a stable airway. In the severely compromised group (patients unable to maintain saturation on room air above 95%, respiratory rate > 25, or a significant airway compromise on fiberoptic laryngoscopy) a definitive airway is required. Awake fiberoptic-assisted intubation should be attempted first; if this fails then a surgical tracheostomy is performed under local anaesthesia.

In the other group, where patients are able to maintain normal oxygen saturation and respiratory rate on room air and where no significant airway compromise is evident on fiberoptic examination, airway is managed conservatively. This involves close airway observation (oxygen saturation, respiratory rate, and serial fiberoptic laryngoscopy) in a high dependency unit (HDU) or ENT ward.

After the initial clinical assessment and airway decision all patients should undergo CT scanning of their neck and thorax for further detailed airway and deep neck spaces evaluation. Any abscess or collection cavity should be drained, and both groups should be kept in an HDU or ENT ward for hourly airway assessment for 24–48 hours.

## 6. Conclusion

Airway management in Ludwig's angina is the gold standard foundation for the management. Recently this evolved from aggressive airway management into a more conservative one. This is based on close airway observation on a specialised airway unit and a serial clinical airway assessment. Improved imaging modalities, antibiotic therapy, surgical skills, and clinical experience are the key factors behind this change in practice.
